# *JCEM Case Reports*: A New Journal for Clinicians

**DOI:** 10.1210/jcemcr/luac001

**Published:** 2022-11-29

**Authors:** William F Young

**Affiliations:** Division of Endocrinology, Diabetes, Metabolism, and Nutrition, Mayo Clinic, Rochester, MN 55905, USA

With this editorial, the Endocrine Society launches *JCEM Case Reports,* a new, open-access, online-only journal. Original case reports impart valuable insights and clinical nuances that cannot be found in large case series, clinical trials, or clinical practice guidelines. Detailed descriptions of individual patients have had a profound impact on medicine over the years—initial discoveries of many disorders have been disseminated through case reports [1–5]. Case reports, some of which were highlighted as practice-changing *Landmark Articles in Medicine* by the American Medical Association, have been and continue to be integral to the advancement of medical knowledge [5]. Case reports can also deepen our understanding of endocrine disorders, enhance clinical skills, and suggest useful areas for research.

We welcome educational clinical cases that are well described, have clear learning points, and are of special significance to early-career endocrinologists and members of endocrine care teams. We are particularly interested in exploring ways to effectively diagnose and treat endocrine conditions in regions with limited clinical resources; these cases may have important implications for a wider audience. We also encourage clinicians to submit case reports on common endocrine disorders with unique diagnostic, ethical, or management challenges. In addition, we are keen to publish cases on rare endocrine disorders that present with a new association, unexpected findings, or unanticipated treatment responses.

We aim for *JCEM Case Reports* to be at the top of the reading list for clinical endocrinologists and endocrine care providers and to become the preferred forum to share challenging cases and disseminate valuable clinical pearls to busy clinicians. We are especially eager to encourage article submissions from aspiring endocrinologists, endocrine trainees, early-career endocrinologists, and all endocrine care providers. Many of my best teaching and learning opportunities have been based on the care of individual patients with perplexing clinical scenarios. When I was an internal medicine resident, my very first clinical publication was a case report describing a patient with a pituitary gland disorder that was a diagnostic conundrum [6]. Caring for that patient and writing the report helped ignite my interest in pursuing a career in endocrinology. Too often, the clinical pearls that we learn from managing difficult clinical situations are never shared. *JCEM Case Reports* will be the forum for clinicians to pass on these valuable clinical insights.

In an effort to make the journal easily accessible to clinicians from around the world, laboratory values will be presented in both Système International (SI) and conventional units. *JCEM Case Reports* is truly an international journal. Our associate editors are from 3 continents and 6 countries. Our editorial board has 185 members from 34 different countries. Recognizing that many article submissions will be from clinicians in training, our editorial team is charged to evaluate the methodologic quality of case reports and work with authors to optimize their submissions with respect to case selection, ascertainment, causality, and reporting [7]. All authors will be required to use a uniform manuscript template that includes tips on how to polish their manuscript. As recommended in the CARE (CAse REport) guidelines, manuscript sections will include an introduction, case presentation, diagnostic assessment, treatment, outcome and follow-up, discussion, and bulleted learning points [8]. The optimal case report is factual, concise, well organized, presented clearly, and easily readable [9].

To bring our audience additional context and guidance, we will recruit experienced clinicians to write commentaries on similarly themed published case reports. In addition to case reports and invited commentaries, the journal will encourage submissions of educational images in endocrinology. Submissions should visually demonstrate endocrine conditions and capture what the clinician sees in the exam room or on a computer imaging system.

*JCEM Case Reports* is launching with 6 issues per year and will operate within a continuous, online publication model. Accepted manuscripts will appear online as *Advance Articles* prior to copyediting and will be citable at that point. After an *Advance Article* has been copyedited and the final version has been approved, it will appear online in an issue. All content will be free for reading worldwide.

We look forward to receiving your case report manuscripts!
